# An elastic traction device for facilitating endoscopic submucosal dissection of a submucosal tumor at the cardia

**DOI:** 10.1055/a-2271-5890

**Published:** 2024-04-11

**Authors:** Yaxuan Cheng, Enqiang Linghu, Bi Ya-Wei, Song Su, Ningli Chai

**Affiliations:** 1 651943 Gastroenterology, Chinese PLA General Hospital First Medical Center, Beijing, China

A common challenge encountered during endoscopic submucosal dissection (ESD) is the lack of traction required to achieve an adequate submucosal view of the dissection area and to avoid mucosal and muscle layer injury, perforation, and bleeding 1 2 . It is even more challenging to perform ESD for tumors located at the cardia of the stomach. Herein, we report a case in which an elastic traction device (Micro-tech, Nanjing, China), consisting of a rotatable tissue clip and an elastic band in the shape of a figure 8 attached to the arm of the clip, was used to improve the intraoperative view and facilitate submucosal tumor (SMT) resection at the cardia. We call this device an “8-shaped band” ( Fig. 1 ).

**Fig. 1:**
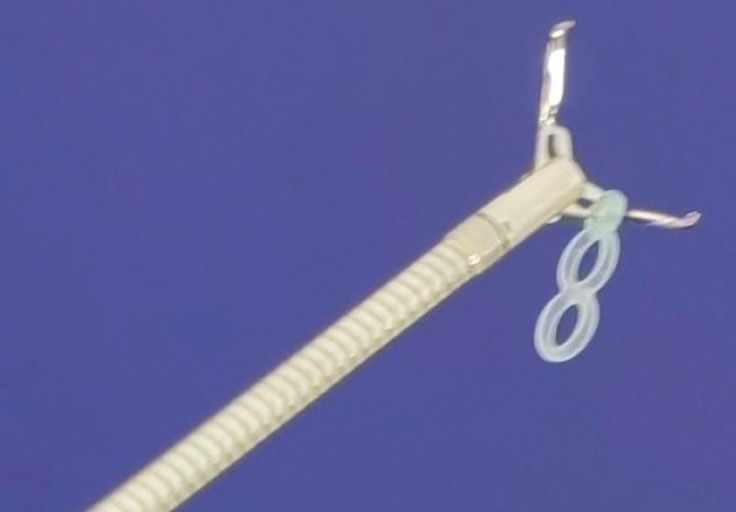
The “8-shaped band”.

A 55-year-old man underwent ESD for an SMT located at the cardia ( Fig. 2 , Video 1 ). Following submucosal injection, the proximal mucosa at the tumor edge was dissected and standard submucosal dissection was performed with an insulated-tip knife (Olympus, Tokyo, Japan). With further dissection, it was difficult to determine the relationship between the tumor root, surface mucosa, and muscular layer ( Fig. 3 ). Therefore, the clip-ring traction device was attached to the tumor root. Then, another clip was hooked to the second loop of the 8-shaped band and was in turn attached to the anterior wall of the gastric corpus. Finally, the target tumor was pulled, and an adequate view of the tumor root was achieved ( Fig. 4 ). En bloc resection was achieved without adverse events ( Fig. 5 ). The clip-ring was removed with forceps to retrieve the resected tumor.

**Fig. 2:**
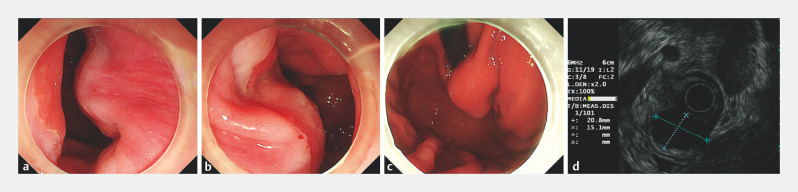
Submucosal tumor located at the cardia. **a** Submucosal lesion causing protrusion at the 3 o’clock position of the cardia. **b** Smooth mucosal surface of the lesion, consistent with normal gastric mucosa. **c** The lesion was observed under a retroflexed view of the gastroscope. **d** Endoscopic ultrasound image of the 20.8 × 15.1-mm lesion, which originated from the muscularis propria and demonstrated a uniform hypoechoic appearance.

**Fig. 3:**
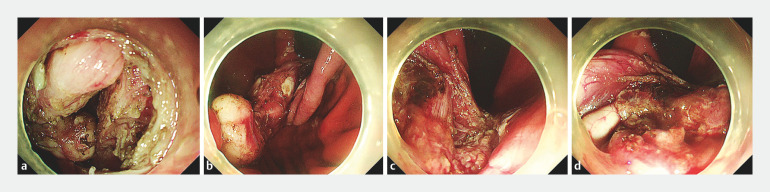
With continuing dissection, adequate traction was difficult to maintain.

**Fig. 4:**
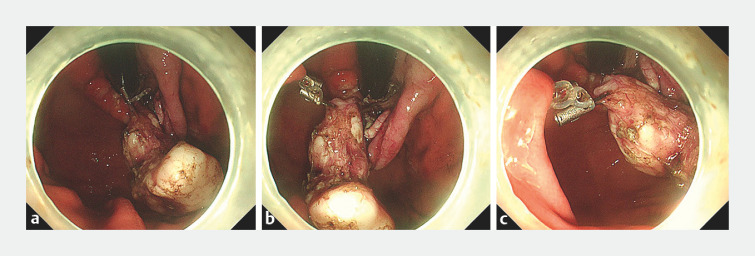
The target tumor was pulled and an adequate view of the tumor was achieved following the deployment of the 8-shaped band device. **a** The clip-ring traction device was attached to the tumor root. **b** Another clip hooked the second loop of the band and was then attached to the anterior wall of the gastric corpus. **c** The target tumor was pulled, and an adequate view of the tumor root was achieved.

**Fig. 5:**
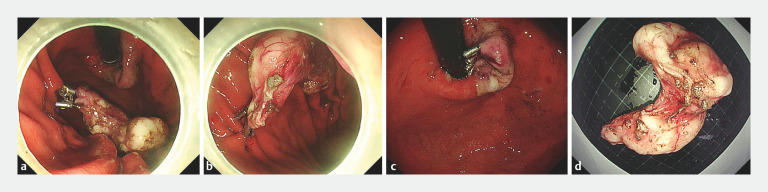
En bloc resection was performed. **a** The clip-ring was cut with forceps to retrieve the resected specimen. **b** The specimen was resected completely, and the resected specimen was kept temporarily in the stomach cavity. **c** Closure of the mucosal defect using endoscopic clips. **d** Macroscopic appearance of the intact specimen.

Endoscopic submucosal dissection (ESD) for an irregularly shaped leiomyoma located at the cardia was performed using an elastic 8-shaped traction device.Video 1

During the late stage of ESD for an SMT, the mucosa begins to shrivel and the tumor is displaced. The presented 8-shaped band enables traction to be applied in any direction and repeated. Furthermore, traction strength can be adjusted using a gas control without the need for endoscope reinsertion. In conclusion, this novel elastic traction device can facilitate ESD for SMTs, especially for tumors located at the cardia.

Endoscopy_UCTN_Code_TTT_1AO_2AG

## Notes

Endoscopy E-Videos https://eref.thieme.de/e-videos
*E-Videos* is an open access online section of the journal *Endoscopy* , reporting on interesting cases and new techniques in gastroenterological endoscopy. All papers include a high-quality video and are published with a Creative Commons CC-BY license. Endoscopy E-Videos qualify for HINARI discounts and waivers and eligibility is automatically checked during the submission process. We grant 100% waivers to articles whose corresponding authors are based in Group A countries and 50% waivers to those who are based in Group B countries as classified by Research4Life (see: https://www.research4life.org/access/eligibility/ ). This section has its own submission website at https://mc.manuscriptcentral.com/e-videos .
